# Supplementary material for: Theoretical Investigation of Delafossite-Cu2ZnSnO4 as a Promising Photovoltaic Absorber
Source: Nanomaterials (Basel). 2023 Dec 10;13(24):3111. doi: 10.3390/nano13243111 (PMC10745759; doi:10.3390/nano13243111)
Supplement: Supplementary file 1 [file nanomaterials-13-03111-s001.zip › nanomaterials-2754319-supplementary.pdf]

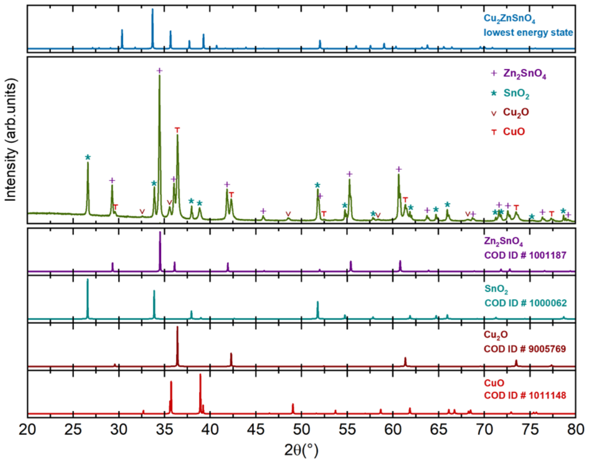

Figure S1. X-ray diffraction (XRD) 2θ scan of a synthesized bulk specimen. High-temperature sintering using an alumina crucible is utilized in this work (dark yellow). Four binary and ternary oxides, Zn_2SnO_4, SnO_2, Cu_2O, and CuO, are formed instead of CZTO. For reference, the simulated powder XRD pattern of delafossite-CZTO (dodger blue) and the experimental powder XRD pattern of Zn_2SnO_4 (purple), SnO_2 (teal), Cu_2O (dark red), and CuO (red), are listed in the figure.

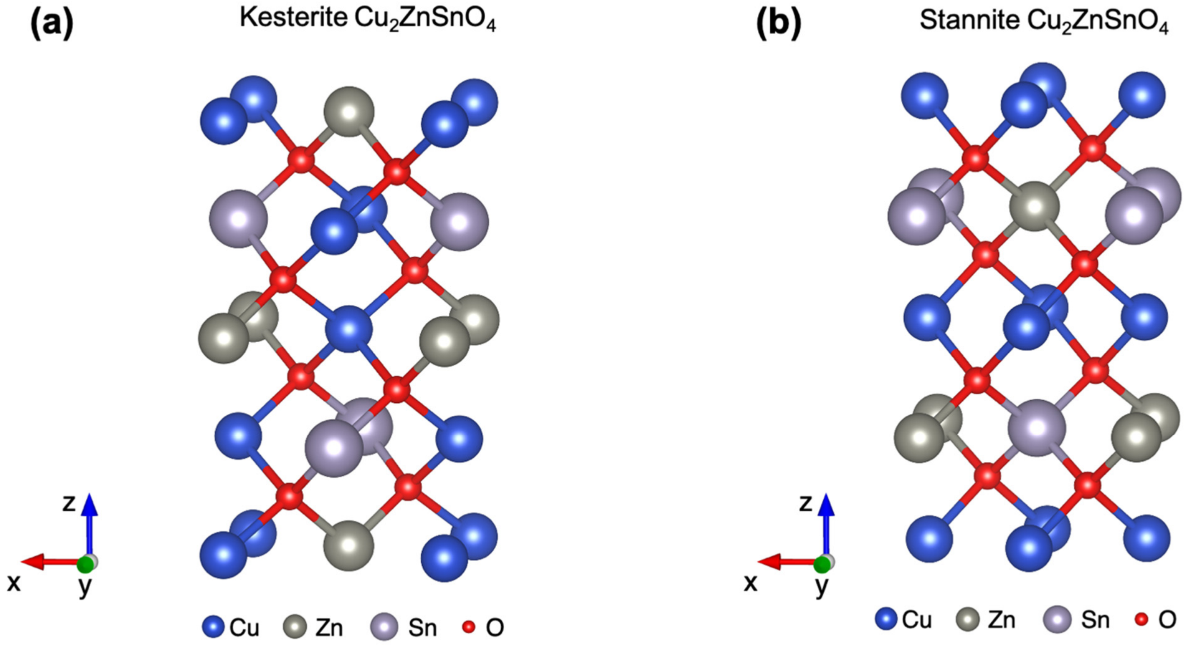

Figure S2. Crystal structure of zincblende-derived Cu_2ZnSnO_4 (CZTO) (a) Kesterite and (b) stannite structure of CZTO. For reference, kesterite and stannite structures are the stable structures for CIGS and CZTS.

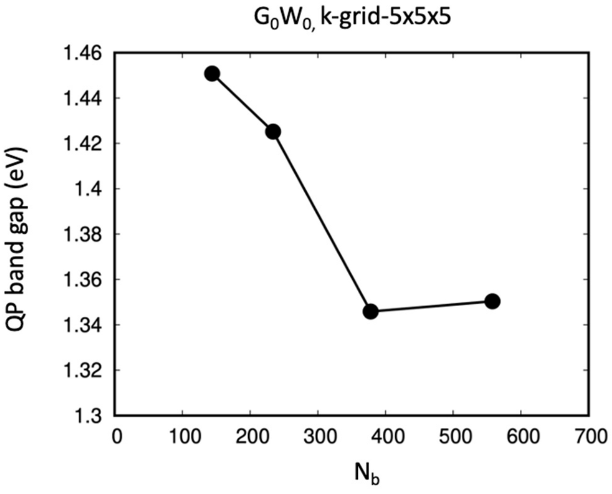

Figure S3. The convergence of the quasiparticle band gap with respect to the number of bands on a 5×5×5 k-grid on Cu_2ZnSnO_4 (CZTO).
